# Pyoderma gangrenosum du cuir chevelu: à propos d’un cas

**DOI:** 10.11604/pamj.2017.26.34.11290

**Published:** 2017-01-23

**Authors:** Békaye Traoré, Youssouf Fofana

**Affiliations:** 1 Service de Dermatologie du Centre National d’Appui à la Lutte Contre la Maladie (CNAM), Bamako (Mali)

**Keywords:** Pyoderma gangrenosum, céphalique, thalidomide, auto greffe cutanée, Pyoderma gangrenosum, cephalic, thalidomide, auto skin grafting

## Image en médecine

Le pyoderma gangrenosum (PG) est une dermatose inflammatoire chronique rare caractérisé par des ulcérations chroniques stériles et récurrentes. Il reste un diagnostic d’exclusion de toutes les autres affections pustulo-ulcéreuses notamment infectieuses. Il est associé dans deux tiers des cas à des affections morbides variées, qui peuvent révéler la maladie. La localisation au cuir chevelu est exceptionnelle et pose un véritable problème de prise en charge. Nous rapportons un cas qui a bien répondu à l’auto greffe cutanée. Il s’agissait d’une patiente de 49 ans aux antécédent de deux cures de myome (2001), qui présentaient depuis 6 mois une ulcération céphalique de 20/15 cm à bordure régulière, légèrement décollée, la pression était douloureuse et faisait sourdre du pus, le fond était peu creusant rouge et nauséabonde. Ailleurs le reste de l’examen clinique était normal. L’ulcération post fasciite nécrosante, lupus discoïde dégénéré, pyoderma gangrenosum furent évoquée comme hypothèses diagnostiques. Les examens complémentaires ont montré une anémie microcytaire hypochrome, une sérologie rétrovirale positive à VIH1, un taux de CD4 à 150/mm^3^, le prélèvement du pus a retrouvé le Staphylococcus Aureus. Le reste du bilan biologique était normal. La patiente n’était pas consentante pour la biopsie. Un traitement antiseptique, antibiotique conformément à l’antibiogramme et antalgique fut instauré pendant deux semaines sans succès après la trithérapie. Le diagnostic de pyoderma gagrenosum fut évoqué, un traitement à base de thalidomide 200mg (400mg par jour). Après un mois le pus et le suintement ont taris. Nous avons effectué trois séances d’auto greffe en pastille. La cicatrisation était obtenue en deux mois.

**Figure 1:**
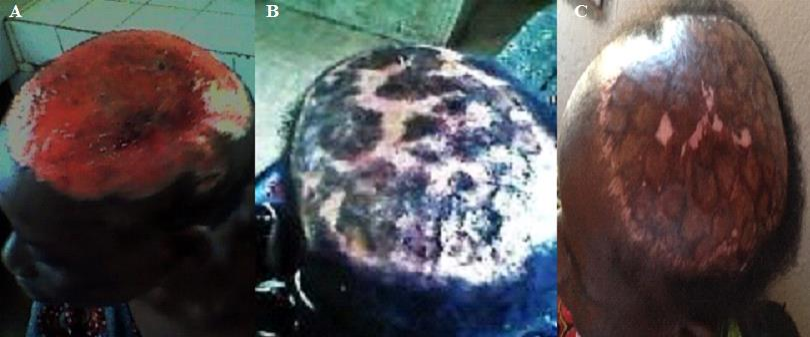
A) large ulcération du cuir chevelu; B) cicatrisation après un mois d’autogreffe; C) 2 mois après la greffe

